# Comparison of Trapezoidal-Shaped 3D Plates and Conventional Miniplates for the Stabilization of Subcondylar Fractures

**DOI:** 10.7759/cureus.114083

**Published:** 2026-08-06

**Authors:** Ranjith Kumar M, Anupriya L, Marimuthu P, Akash Ganguly, Ghulam S Hashmi, Sajjad A Rahman, Nithya Bakialakshmi K

**Affiliations:** 1 Oral and Maxillofacial Surgery, N.R.K. Dental Clinic, Salem, IND; 2 Oral and Maxillofacial Surgery, Dr. Ziauddin Ahmad Dental College and Hospital, Aligarh Muslim University (AMU), Aligarh, IND; 3 Oral and Maxillofacial Surgery, All India Institute of Medical Sciences (AIIMS)-Central Armed Police Forces Institute of Medical Sciences (CAPFIMS), New Delhi, IND; 4 Oral and Maxillofacial Surgery, Indira Gandhi Institute of Medical Sciences, Patna, IND; 5 Periodontology, K.V.G. Dental College and Hospital, Sullia, IND

**Keywords:** mandibular condyle, mandibular fracture fixation, open reduction and internal fixation, retromandibular approach, trapezoidal 3d plate

## Abstract

Objectives

This study aimed to compare trapezoidal-shaped 3D plates and conventional miniplates for the stabilization of subcondylar fractures. The primary outcome measure was postoperative occlusal outcome; secondary outcome measures included operative time, functional recovery (maximum mouth opening and lateral mandibular movement), and postoperative complications.

Materials and methods

A prospective study was conducted on 20 patients diagnosed with subcondylar fractures in the Department of Oral and Maxillofacial Surgery, Dr. Ziauddin Ahmad Dental College and Hospital, Faculty of Medicine, Aligarh Muslim University, India. All patients underwent open reduction and internal fixation (ORIF) via the retromandibular approach. The patients were divided into two groups: one group was treated with trapezoidal-shaped 3D plates, while the other received conventional miniplates. The time taken for plate fixation, postoperative occlusion, functional outcomes (maximum mouth opening and lateral mandibular movements), and complications were assessed and compared between the groups.

Results

Both groups achieved satisfactory anatomical reduction confirmed by postoperative orthopantomogram and Townes view radiographs. The trapezoidal 3D plate group demonstrated significantly superior postoperative occlusal outcomes at all follow-up time points. By the fourth week, 100% of Group I patients achieved satisfactory occlusion versus 50% in Group II. Mouth opening and lateral mandibular movements were comparable between groups at three months. The mean operative time was significantly shorter in Group I (21.80 min vs. 40.20 min; p < 0.0001). Complication rates were minimal and comparable between groups.

Conclusion

Trapezoidal-shaped 3D plates demonstrated superior postoperative occlusal stability and reduced operative time compared to conventional double miniplates in the management of mandibular subcondylar fractures, while providing comparable functional and radiographic outcomes. Larger studies with longer follow-up are warranted to validate these findings.

## Introduction

Mandibular condylar fractures account for 17.5-52% of all mandibular fractures [1]. The most common etiology is direct trauma; however, it may also occur as a result of indirect forces [2,3]. Treatment options for condylar fractures are based on clinical and imaging characteristics of the fracture, extent of injury, whether unilateral or bilateral, the anatomical level of fracture, degree of displacement and dislocation, the size and position of the fractured condylar segment, and the deranged occlusion, among others [4,5]. As in the literature, three main treatments are advocated for adult condylar fractures: closed reduction with maxillomandibular fixation (MMF) followed by functional rehabilitation, functional therapy without MMF, and open reduction with or without MMF. Any injury of the condyle deserves special consideration owing to its complex anatomy, biomechanical behavior, and healing potential. According to Schneider et al., fractures with a deviation of more than 10° or a shortening of the ascending ramus of more than 2 mm should be treated with open reduction and fixation, regardless of the level of the fracture [6]. It raises a problem of finding a fixation device that can resist local strains, adapting to the anatomical and functional peculiarities of the region [7], and also providing easy surgical access while being cost-effective to the patient; a miniature osteosynthesis device becomes essential for stabilization of subcondylar fractures because of the usually small size of condylar fragments. It is also mandatory to place these plates along the “ideal line of osteosynthesis’’ for dictating a predictable outcome. Champy et al. experimentally located these strain lines in the mandibular body, symphysis, and angle region [8]. Later, Meyer et al. proposed these lines in the condylar region [9]. Although the widely accepted technique for osteosynthesis of subcondylar fractures was the double-miniplate technique, trapezoidal condylar plates were developed to meet biofunctional demands in the condylar region, which was first applied by Meyer et al. [10,11]. It was hypothesized that trapezoidal-shaped 3D plates provide better stability and improved clinical outcomes compared to conventional miniplates in the management of subcondylar fractures.

This prospective comparative study was conducted to evaluate the clinical results of the trapezoidal-shaped 3D plate for stabilization of subcondylar fractures. The primary objective was to compare postoperative occlusal outcomes between the trapezoidal 3D plate and conventional double miniplates. Secondary objectives were to compare the two techniques with respect to operative time, functional recovery (maximum interincisal distance and lateral mandibular movement), and postoperative complication rates.

## Materials and methods

This prospective comparative study was conducted in the Department of Oral and Maxillofacial Surgery, Dr. Ziauddin Ahmad Dental College and Hospital, Faculty of Medicine, Aligarh Muslim University, India. Patients presenting to the outpatient department with mandibular subcondylar fractures for whom open reduction and internal fixation (ORIF) was indicated were considered for inclusion in the study. Inclusion criteria comprised subcondylar fractures with ramus height shortening and impaired occlusion, inability to achieve satisfactory occlusion by closed reduction, lateral dislocation of the condyle, and fractures involving foreign body invasion. Patients with condylar head fractures, age below 18 years, systemic contraindications to open reduction, and those unable to provide consent were excluded.

**Figure 1:**
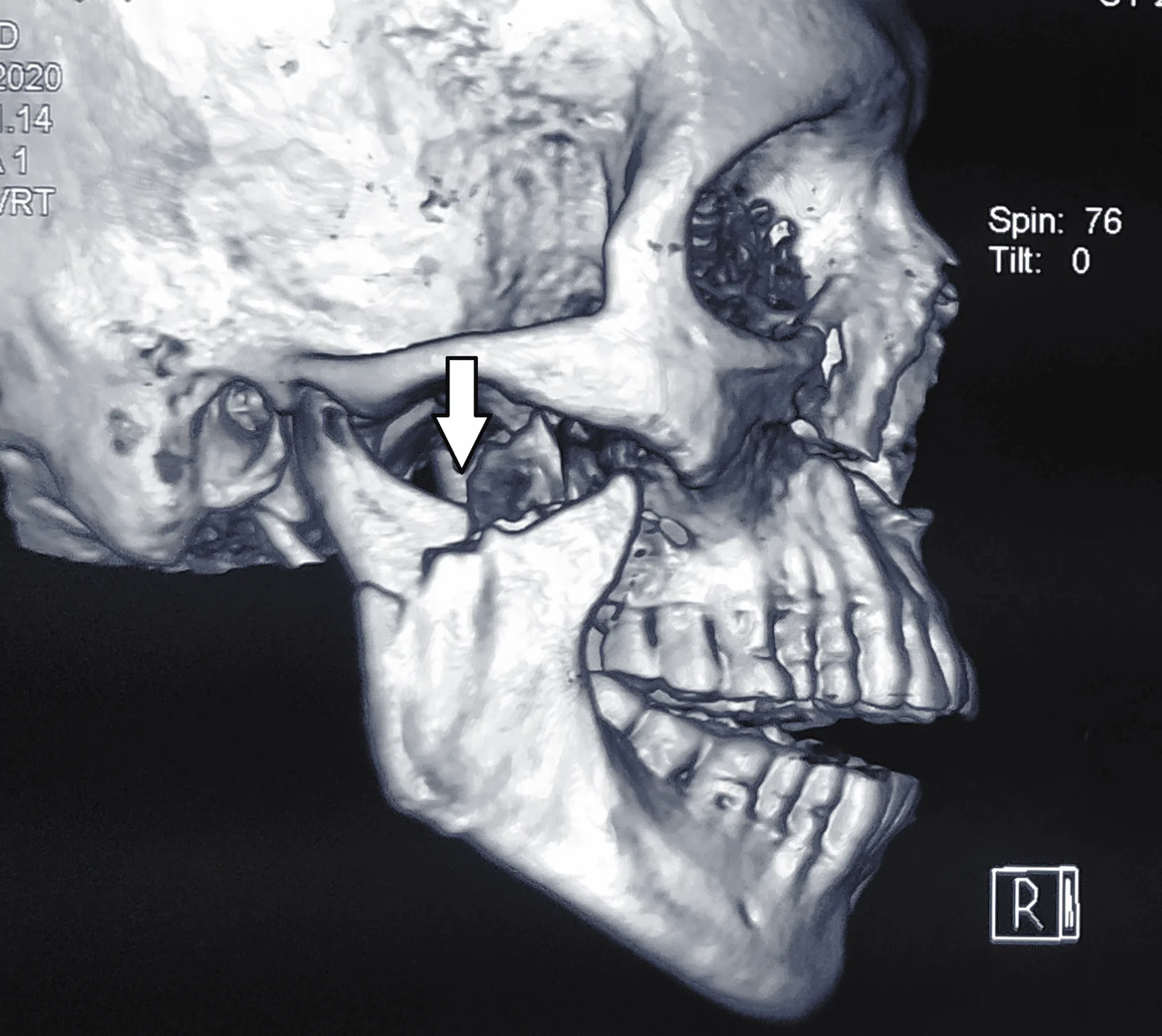
Three-dimensional computed tomography showing subcondylar fracture displacement depicted by the white arrow.

A total of 20 patients who satisfied the eligibility criteria were enrolled. The diagnosis of subcondylar fracture was established based on detailed history, thorough clinical examination, and radiographic evaluation. Orthopantomogram was obtained in all cases, and additional computed tomography (CT) or three-dimensional CT scans without contrast, including axial, coronal, and sagittal views, and 3D reconstruction, were advised whenever necessary to accurately assess fracture displacement and anatomical configuration (Fig. 1).

Prior to commencement of the study, approval was obtained from the Institutional Review Board (IRB) of the institution, and the study protocol adhered to the ethical principles outlined in the Declaration of Helsinki. Clinical trial registration was also completed before patient recruitment began (Clinical Trials Registry- India (CTRI): CTRI/2019/09/012345). Written informed consent was obtained from all participants prior to their inclusion in the study.

Group allocation was performed on a quasi-experimental basis: the first 10 consecutive eligible patients were assigned to Group I (3D trapezoidal plate) and the subsequent 10 patients to Group II (conventional miniplates). This sequential, non-randomized method of allocation is acknowledged as a methodological limitation of the study, as it introduces the possibility of selection bias. A formal a priori power calculation was not performed; post-hoc analysis indicates that with a sample size of n = 10 per group, the study has approximately 60-65% power to detect large effect sizes (Cohen's d ≥ 0.8) at α = 0.05, which limits the ability to draw definitive conclusions and should be considered when interpreting the results.

Of the 20 patients included, 18 presented with isolated unilateral subcondylar fractures, while the remaining two had a concurrent mandibular symphysis fracture that was stabilized simultaneously during the same operative procedure. Subcondylar fractures were classified according to the AO CMF (AO craniomaxillofacial) classification system for condylar process fractures, which stratifies fractures by anatomical level (condylar head/diacapitular, condylar neck, and condylar base/subcondylar), degree of fragmentation, and displacement or dislocation of the fragment relative to the fossa. Only fractures classified as subcondylar (condylar base) on this system were included in the study, in line with the exclusion criteria stated above. The two groups were comparable with respect to age (Group I: 31.4 ± 6.8 years; Group II: 29.7 ± 5.9 years; p = 0.52), sex distribution (Group I: 7 male, 3 female; Group II: 6 male, 4 female; p = 0.63), and smoking status (Group I: 2 smokers; Group II: 3 smokers; p = 0.62), with no statistically significant intergroup differences in baseline demographic characteristics.

**Figure 2:**
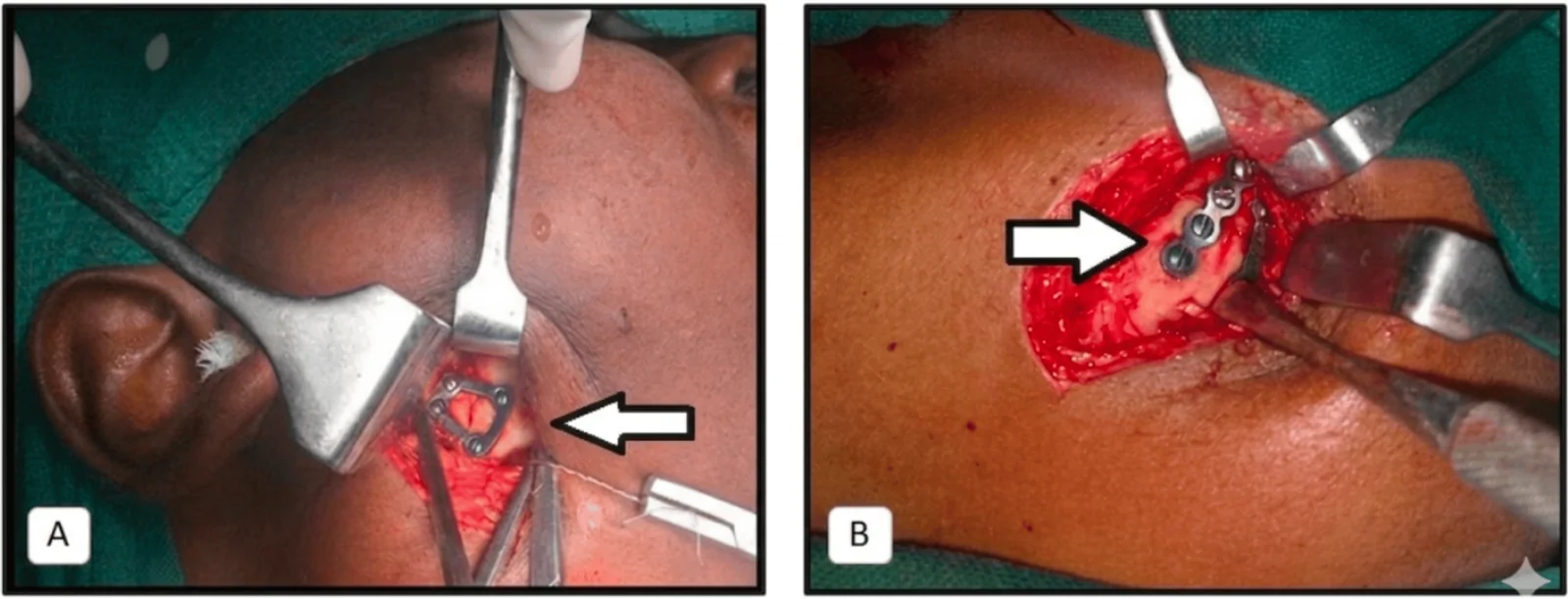
A) Intraoperative image of the trapezoidal 3D titanium plate. B) Conventional miniplates. Both the plates have been indicated with white arrows.

All surgical procedures were performed under general anesthesia by the same team of surgeons to minimize operator variability. A standard retromandibular approach was used in all cases to expose the fracture site. Following anatomical reduction of the fractured segments, fixation was carried out according to group allocation. In Group I, stabilization was achieved using a 2.0-mm titanium 3D trapezoidal condylar miniplate with four holes. The plate was secured using 2.0-mm × 6-mm monocortical titanium screws, with two screws placed on either side of the fracture line to ensure stable three-dimensional fixation along the ideal lines of osteosynthesis (Fig. 2A). In Group II, fixation was performed using two 2.0-mm conventional titanium miniplates (four holes without gap). Each plate was secured with 2.0-mm × 6-mm monocortical titanium screws (Fig. 2B). One plate was positioned parallel to the posterior border of the ramus, while the second plate was placed just posterior and inferior to the sigmoid notch, ensuring adequate stabilization across the fracture line. After confirmation of stable fixation and hemostasis, layered wound closure was performed in all cases.

Postoperative MMF was not used routinely in this study, as all patients achieved satisfactory occlusion at the time of wound closure under general anesthesia, and early mobilization has been shown to optimize functional rehabilitation following ORIF of condylar fractures. Elastic guidance was provided selectively in cases where intraoperative occlusal fine-tuning was required.

**Figure 3:**
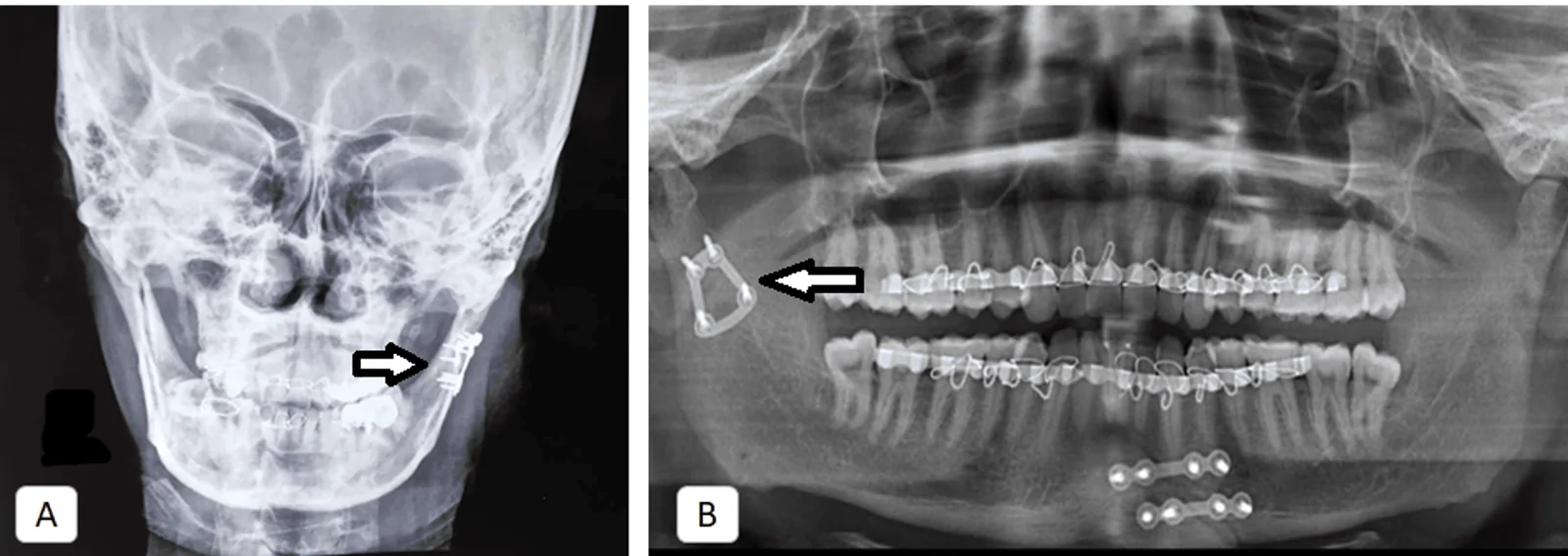
A) Postoperative Townes’ view with miniplates. B) Postoperative OPG with the trapezoidal plate at the condyle region depicted with white arrows.

Postoperative radiographic evaluation using orthopantomogram and Townes view was performed on the first postoperative day to assess adequacy of reduction and fixation. Operative time was measured from the point of fracture exposure (i.e., after completion of the retromandibular approach and identification of the fracture site) to the completion of final screw tightening and confirmation of stable fixation; this definition was standardized across all cases. Patients were subsequently followed up at the first week, second week, fourth week, and third postoperative month (Fig. 3). At each follow-up visit, clinical evaluation included assessment of postoperative occlusion, maximum interincisal distance (maximal mouth opening), lateral mandibular movements, postoperative pain, and any complications such as infection, bleeding, or facial nerve weakness. All observations were systematically recorded.

All postoperative clinical and radiographic assessments were performed by the same evaluating surgeon at each follow-up visit. Given the visible difference between the trapezoidal 3D plate and the double miniplate construct on both clinical inspection and radiographic imaging, formal blinding of the outcome assessor to group allocation was not feasible and was not performed. This represents an additional limitation of the study design and has been acknowledged accordingly.

Occlusal outcomes were graded using the following standardized criteria: "Achieved" - restoration of the patient's pre-injury occlusion with complete intercuspation and no clinically detectable malocclusion; "Satisfactory" - minor occlusal discrepancy (≤2 mm) not requiring further intervention and resolving with functional exercises; and "Deranged" - clinically significant malocclusion (>2 mm open bite, crossbite, or shift) requiring active management.

The collected data were compiled and analyzed using IBM SPSS Statistics for Windows, version 16.0 (released 2007, IBM Corp., Armonk, NY). Descriptive statistics including mean, standard deviation, and frequency distribution were calculated. The normality of data distribution was assessed using the Shapiro-Wilk test and was found to be normally distributed. Quantitative variables were compared between the two groups using the independent t-test, while qualitative variables were analyzed using Pearson's Chi-square test. A 95% confidence interval and a significance level of 5% (p < 0.05) were applied for statistical analysis. All classification systems and assessment tools used in this study are publicly available and free for academic use.

## Results

Various clinical and radiographic parameters were assessed and recorded at predetermined postoperative intervals.

Postoperative occlusion

Postoperative occlusion was significantly better in Group I (trapezoidal 3D plate) compared to Group II (conventional miniplates) at multiple follow-up intervals. On the first postoperative day, satisfactory occlusion was achieved in 60% of patients in Group I compared to 20% in Group II (p = 0.005). At the first week, 60% of patients in Group I demonstrated satisfactory occlusion versus 20% in Group II (p = 0.019). By the second week, 90% of patients in Group I achieved satisfactory occlusion compared to 20% in Group II (p = 0.002). At the fourth week, 100% of patients in Group I showed satisfactory occlusion, whereas only 50% of patients in Group II achieved the same (p = 0.010). At the third postoperative month, satisfactory occlusion was observed in 90% of patients in Group I compared to 50% in Group II (p = 0.010) (Table 1). These findings indicate that the trapezoidal 3D plate provides superior postoperative occlusal stability compared to conventional miniplates.

**Table 1:** Postoperative occlusion at different time intervals Test applied: Pearson's Chi-square test. Significance level: p < 0.05.

Time interval	Category	Group I (n = 10)	Group II (n = 10)	χ² (df)	p-value
1st post-op day	Achieved	6 (60%)	2 (20%)	10.444 (2)	0.005
	Satisfactory	3 (30%)	0 (0%)		
	Deranged	1 (10%)	8 (80%)		
1st week	Achieved	8 (80%)	2 (20%)	7.886 (2)	0.019
	Satisfactory	2 (20%)	5 (50%)		
	Deranged	0 (0%)	3 (30%)		
2nd week	Achieved	9 (90%)	2 (20%)	9.899 (1)	0.002
	Satisfactory	1 (10%)	8 (80%)		
4th week	Achieved	10 (100%)	5 (50%)	6.667 (1)	0.010
	Satisfactory	0 (0%)	5 (50%)		
3rd month	Achieved	9 (90%)	5 (50%)	6.667 (1)	0.010
	Satisfactory	1 (10%)	5 (50%)		

Maximum interincisal distance

The mean maximum interincisal distance (MID) at the fourth postoperative week was significantly higher in Group I (36.60 mm) than in Group II (33.20 mm) (p = 0.033). However, no statistically significant differences were observed between the groups on the first postoperative day, first week, second week, or at the third month (Table 2). The overall mean maximal active interincisal opening in both groups at the end of the observation period was 37 mm (range: 30-60 mm; SD ± 2.1 mm), indicating comparable functional recovery.

**Table 2:** Comparison of mean functional parameters and operative time (independent t-test) Test applied: independent samples t-test. df = 18.

Variable	Time interval	Group I mean	Group II mean	t value (df = 18)	p-value
Maximum interincisal distance (mm)	1st day	21.70	20.10	0.67	0.51
	1st week	28.40	24.50	1.64	0.12
	2nd week	33.30	29.60	1.74	0.10
	4th week	36.60	33.20	2.30	0.033
	3rd month	37.80	37.00	0.82	0.42
Lateral mandibular movement (mm)	1st day	9.00	9.30	0.60	0.55
	1st week	10.20	10.00	0.44	0.67
	2nd week	11.10	10.40	1.27	0.22
	4th week	11.70	11.00	1.38	0.19
	3rd month	12.00	11.20	1.60	0.13
Operative time (minutes)	Intraoperative	21.80	40.20	8.52	0.0001

Lateral mandibular movements

The mean lateral mandibular movement in both groups ranged between 11 and 12 mm, which falls within the normal physiological range. By the third postoperative month, no statistically significant difference was observed between Group I and Group II (Table 2), suggesting comparable restoration of lateral mandibular function.

Postoperative radiographic evaluation

Orthopantomographic and Townes views were obtained on the first postoperative day to evaluate anatomical reduction in both anteroposterior and mediolateral dimensions (Figures 3A, 3B). Radiographs were assessed by a single observer who assigned scores accordingly. No statistically significant difference was found between the two groups in terms of proper anatomical reduction (p = 0.329) (Table 3).

**Table 3:** Postoperative complications and radiographic evaluation (chi-square test) Test applied: Pearson’s chi-square test

Parameter	Group I (n = 10)	Group II (n = 10)	χ² (df)	p-value
Post-operative infection	0 (0%)	0 (0%)	NA	NA
Post-operative bleeding	0 (0%)	0 (0%)	NA	NA
Temporary paraesthesia	1 (10%)	0 (0%)	1.053 (1)	0.305
Radiographic anatomic reduction (satisfactory)	8 (80%)	6 (60%)	0.952 (1)	0.329
Pain – 1st post-op day (pain-free)	1 (10%)	5 (50%)	3.810 (1)	0.051
Pain – 1st week (pain-free)	10 (100%)	6 (60%)	5.000 (1)	0.025
Pain – 2nd week onward (pain-free)	10 (100%)	10 (100%)	NA	1.00

Mean operative time

The mean operative time was significantly shorter in Group I (21.80 minutes) compared to Group II (40.20 minutes), with a mean difference of 18.40 minutes (p = 0.000) (Table 2). In addition, intraoperative observation revealed that adaptation of the 3D trapezoidal plate along the ideal lines of osteosynthesis in the subcondylar region was technically easier compared to conventional miniplates.

Postoperative complications

No cases of postoperative wound infection were observed in either group throughout the follow-up period; hence, statistical comparison was not applicable. Similarly, no patient in either group experienced postoperative bleeding at any time interval (Table 3).

With respect to neurosensory disturbance, one patient in Group I exhibited temporary weakness of the marginal mandibular branch of the facial nerve, evident during active mouth puckering and downward movement of the affected corner of the mouth. The weakness resolved completely within two months, and full recovery was noted by the third postoperative month. The difference between groups was not statistically significant (p > 0.05).

Postoperative pain was evaluated at different follow-up intervals. A statistically significant difference was observed only at the first postoperative week, where 100% of patients in Group I were pain-free compared to 60% in Group II (p = 0.025). At other time intervals, no statistically significant differences were observed between the groups (Table 3). By the end of the third postoperative month, all patients in both groups were pain-free during maximum mouth opening, lateral excursions, and protrusive movements.

## Discussion

ORIF of condylar fractures remains one of the most debated topics in maxillofacial surgery, particularly in cases associated with loss of vertical ramus height and displacement of the disco-condylar unit, which are widely accepted indications for surgical intervention [12]. The primary objectives of management include restoration of pain-free mandibular function, stable occlusion, and adequate range of mandibular movements [13]. Achieving these goals requires not only an appropriate surgical approach but also the selection of a fixation system capable of withstanding functional loading in the anatomically and biomechanically complex condylar region.

Previous biomechanical investigations have supported the use of double monocortical miniplates, demonstrating superior stability when compared with other plating techniques for condylar fractures [14]. Nevertheless, despite being widely accepted, the double miniplate technique is frequently associated with technical challenges, including restricted visualization of the condylar neck and difficulty in precise plate adaptation, which may compromise anatomical reduction. Reported failure rates of up to 35%, including plate fracture and loosening, further underscore these limitations [15,16]. In response to these concerns, trapezoidal 3D plates were designed based on in-vitro analyses of load distribution, strain patterns, and bone deformation in the condylar region, aiming to provide functionally stable osteosynthesis through geometric configuration [17,18]. Experimental validation has demonstrated that trapezoidal condylar plates are capable of resisting physiological strains and fulfilling the principles of stable fixation in this region [19]. Clinical evaluation has also suggested that 3D trapezoidal plates offer satisfactory rigidity and stability in adult subcondylar fractures managed by open reduction [20].

In the present study, both fixation systems achieved acceptable functional and radiographic outcomes. However, trapezoidal-shaped 3D plates demonstrated significantly better postoperative occlusal results and reduced operative time, likely attributable to easier adaptation along the ideal lines of osteosynthesis and reduced need for multiple plate positioning. Restoration of occlusion is a critical determinant of treatment success, and although literature reports variable rates of postoperative malocclusion, no persistent occlusal discrepancies were observed at follow-up in our series, consistent with previous findings on occlusal outcomes following open treatment of condylar fractures [21,22]. Maximal interincisal opening and lateral mandibular movements were comparable between groups and remained within physiologically acceptable limits, indicating satisfactory functional recovery of the temporomandibular joint [22,23]. Radiographic assessment confirmed accurate anatomical reduction in both groups, which may be attributed to the standardized surgical approach and operative expertise [24,25].

Research conducted by Palani et al. [26] highlights that the geometric configuration of 3D plates facilitates seamless adaptation within the subcondylar region, specifically when aligned with the natural lines of osteosynthesis. Furthermore, the efficacy of fracture reduction following the application of these plates was categorized as excellent to favorable, a finding that resonates with the observations documented by Meyer et al. [21]. Our current investigation mirrors these results, as we observed that the unique design of trapezoidal plates offered superior ease of adaptation and structural alignment when addressing subcondylar fractures. The significantly reduced operative time in Group I (18.4 min mean difference; p < 0.0001) carries practical clinical value, as shorter anaesthesia and operative time are associated with reduced perioperative risk and improved resource utilisation in high-volume centres.

Despite these encouraging findings, certain limitations must be acknowledged. First, the sample size was relatively small, with only ten patients per group, providing approximately 60-65% power to detect large effect sizes (Cohen's d ≥ 0.8) at α = 0.05; this inherently limits statistical power, reduces the likelihood of detecting subtle intergroup differences, and necessitates cautious interpretation of the results. The modest recruitment can be attributed to the strict inclusion criteria, the relative infrequency of isolated subcondylar fractures requiring open reduction within the study period, and the single-center design. Second, group allocation was not randomized but performed sequentially, introducing the possibility of selection bias. Third, the follow-up period was limited to three months, precluding evaluation of long-term hardware complications, condylar remodeling, and late occlusal changes; extended longitudinal assessment would provide a more comprehensive understanding of stability and temporomandibular joint function over time. Fourth, the use of different postoperative imaging modalities across groups (Townes view vs. orthopantomogram) limits radiographic comparability between fixation techniques. Fifth, formal blinding of the outcome assessor to group allocation was not performed; given the visibly distinct appearance of the trapezoidal 3D plate and the double miniplate construct on both clinical and radiographic examination, assessor blinding was not feasible in this study design, which may introduce observer bias in the assessment of subjective outcomes such as occlusal grading and pain scoring.

Limitations

Overall, while the present study suggests potential clinical advantages of trapezoidal 3D plates, the small sample size, non-randomized design, short follow-up period, and heterogeneous postoperative imaging warrant cautious interpretation of the results. These findings should be regarded as preliminary evidence supporting the non-inferiority - and potential superiority in specific parameters - of the 3D trapezoidal plate over conventional miniplates, pending validation in a larger, randomized controlled trial with long-term follow-up.

## Conclusions

The trapezoidal plate group showed significantly better postoperative occlusal outcomes and reduced operative time, while maintaining comparable results in terms of maximal mouth opening, lateral mandibular movements, anatomical reduction, and postoperative complication rates. These findings are consistent with existing evidence supporting ORIF as the preferred treatment for displaced subcondylar fractures and further support the trapezoidal 3D plate as a biomechanically favorable alternative to the conventional double-miniplate technique. The geometric configuration of the trapezoidal 3D plate is based on established principles of three-dimensional osteosynthesis and standardized biomechanical testing and appears to provide enhanced stability and ease of adaptation along the ideal lines of osteosynthesis, thereby facilitating efficient fixation in the anatomically complex subcondylar region.

Although both fixation methods were clinically effective, trapezoidal-shaped 3D plates may be considered a more efficient and functionally stable alternative to conventional miniplates for subcondylar fracture stabilization. These findings should be interpreted in the context of the study's small sample size and non-randomized design; studies with a larger cohort, randomized allocation, and longer follow-up period are required to further validate and generalize the findings of the present study.
